# Management of high-risk and advanced basal cell carcinoma

**DOI:** 10.1007/s12094-014-1272-9

**Published:** 2015-02-03

**Authors:** S. Puig, A. Berrocal

**Affiliations:** 1 Melanoma Unit, Dermatology Department, Hospital Clínic Barcelona, IDIBAPS, University of Barcelona, Villarroel 170, 08036 Barcelona, Spain; 2 Centro Investigación Biomédica en Red en Enfermedades Raras (CIBERER), Barcelona, Spain; 3 Oncology Service, Hospital General Universitario de Valencia, Valencia, Spain

**Keywords:** Basal cell carcinoma, High-risk BCC, Locally advanced BCC, Metastatic BCC, Mohs micrographic surgery, Hedgehog pathway inhibitors

## Abstract

Despite that basal cell carcinoma (BCC) is curative in the vast majority of cases, some patients are at high risk of recurrence and, in a few patients, lesions can progress to a point unsuitable for local therapy and prognosis is quite poor. The aim of the present work is to review clinical and pathologic characteristics as well as classical and new treatment options for high-risk, metastatic and locally advanced BCC. Surgery and radiotherapy remain the selected treatments for the majority of high-risk lesions. However, some patients are located on a blurry clinical boundary between high-risk and locally advanced BCC. Treatment of these patients is challenging and need an individualized and highly specialized approach. The treatment of locally advanced BCC, in which surgery or radiotherapy is unfeasible, inappropriate or contraindicated, and metastatic BCC has changed with new Hedgehog pathway inhibitors of which vismodegib is the first drug approved by FDA and EMA.

## Introduction

Basal cell carcinoma (BCC) is the most common malignant tumor of the skin and is also the most common human malignancy. The incidence of BCC is increasing in many countries around the world. The underlying cause of BCC is unknown, but ultraviolet light exposure and genetic predisposition seem to be the most significant etiological factors [1]. Aging of the population and frequent exposure to sunlight may explain the worldwide increase that has been observed in the incidence of this malignancy [2].

The goal of treatment of BCC according to the National Comprehensive Cancer Network (NCCN) is cure and maximal preservation of function and cosmetics [3]. Early treatment of BCC is curative in the vast majority of cases, thus preventing progression with local approaches such as surgical excision, radiotherapy, topical imiquimod, or photodynamic therapy [4]. The likelihood of recurrence following treatment is used to categorize lesions as low or high risk. Thus, high-risk BCC denotes primary or already relapsed tumors with a significant risk of further relapse after local treatment. The overall 5-year recurrence rate has been estimated to be around 4–5 % [5]. Surgery and radiotherapy are the treatment of choice for most patients with high-risk lesions [6].

Despite that advanced disease is rare, BCC can progress to a point unsuitable for local therapy and prognosis for these patients is quite poor. New treatment options for BCC that inhibit the Hedgehog pathway have led to a redefinition of advanced BCC that includes metastatic BCC and locally advanced BCC [7, 8]. Metastatic BCC is extremely uncommon, with an incidence ranging from 0.0028 to 0.5 % [9]. The most common areas of metastasis are lymph nodes, lungs, bone and parathyroid glands [10]. In turn, locally advanced BCC refers to lesions that are not appropriate for surgery, with medical contraindications for surgery or for whom surgery would result in substantial morbidity or deformity according to the treating physician. The aim of the present work is to review clinical and pathologic characteristics as well as classical and new treatment options for high-risk, metastatic and locally advanced BCC.

### High-risk BCC

Treatment decisions in patients with BCC are usually made on the basis of an estimate of the risk of recurrence. This estimation takes into account clinical and pathologic prognostic factors associated with a high risk of aggressive behavior and a high risk of tumor relapse after primary treatment with curative intent. Firstly, it should be noted that some important clinical features, such as age, duration of lesion and gender do not define high risk of relapse [11]. On the other hand, clinical features defining high risk of relapse include infiltrative growth margins, size, tumor location, histological subtype, recurrent-refractory tumors and previous history of radiotherapy.

Aggressive, infiltrating tumors are frequently ulcerated and have ill-defined margins [12]. In turn, ulcerated BCC is usually larger than non-ulcerated tumors and may be locally destructive. A size larger than 3 cm has been described as a high-risk feature [13]. Notwithstanding the foregoing, this risk factor has been more accurately defined as 1 cm for head and neck tumors and more than 2 cm in other body areas [11]. Tumor location is important as a prognostic factor and a classification in three groups has been proposed accordingly. Thus, trunk and limbs are considered low-risk areas, forehead, cheek, chin, scalp and neck are intermediate-risk areas and, finally, nose and periorificial areas are high-risk areas [11]. Histological subtype should also be taken into account to establish the risk of relapse. The morpheaform, the sclerosing, the infiltrating, the micronodular and the metatypical subtypes are associated with higher risk of relapse [3, 4, 11, 12, 14, 15] as compared to the risk associated with the superficial and the nodular forms. Perineural invasion has prognostic value and its presence is associated with a higher risk of relapse [11, 12]. In contrast, vascular invasion seems to be of no importance. There is no agreement on the prognostic significance of other factors such as a previous history of radiotherapy, for which a retrospective study found an association [16] whereas others consider this issue as a controversial one [11]. Nevertheless, it is considered that more than 30 % of BCCs have mixed pathology, combining less and more aggressive subtypes (i.e., nodular BCC with areas of infiltrating BCC) [12].

Treatment of high-risk BCC includes several options associated with different levels of aggressiveness. Therefore, it is of the utmost importance to ascertain which patients should be treated with an aggressive approach and which patients may perform well with a less invasive treatment. This decision should be taken on the basis of an evaluation of both the performance status of the patient and the risk of relapse. Table 1 summarizes recommendations from major clinical guidelines to treat high-risk BCC.

**Table 1:** Management of high-risk BCC. Recommendations from clinical guidelines

Guideline, year (references)	Recommendations
Sterry 2006 [15]	High-risk positive margins or tumor >2 cm in high-risk site: MMS
	High risk with cosmetic concerns or fragile patients: photodynamic therapy, imiquimod, radiotherapy
	Recurrent tumors: >2 cm or infiltrative or high-risk site: MMS
Dandurand 2006 [11]	High-risk tumors or previous incomplete resection: MMS when available
	Multidisciplinary team decision in selected cases
Telfer 2008 [6]	High-risk positive margins or tumor >2 cm in high-risk site: MMS
	High risk with cosmetic concerns or fragile patients: photodynamic therapy, imiquimod, radiotherapy
	Recurrent tumors: >2 cm or infiltrative or high-risk site: MMS
Connolly 2012 [19]	MMS for primary high-risk and recurrent tumors
	MMS for nodular tumors in H and M areas (see text)
NCCN 2013 [3]	High risk with positive margins: MMS
	Radiotherapy for advanced and non-surgical candidates

*MMS* Mohs micrographic surgery

A non-surgical approach of high-risk BCC may be the best option for a well-defined subset of patients. Indeed, patients who refuse surgery, those who are elderly or patients with poor general health can be best managed with radiotherapy or other local less aggressive treatments. Moreover, local treatment of recurrent non-aggressive (nodular or superficial types) BCC is controversial [17] and these tumors may not require an aggressive approach.

**Fig. 1:**
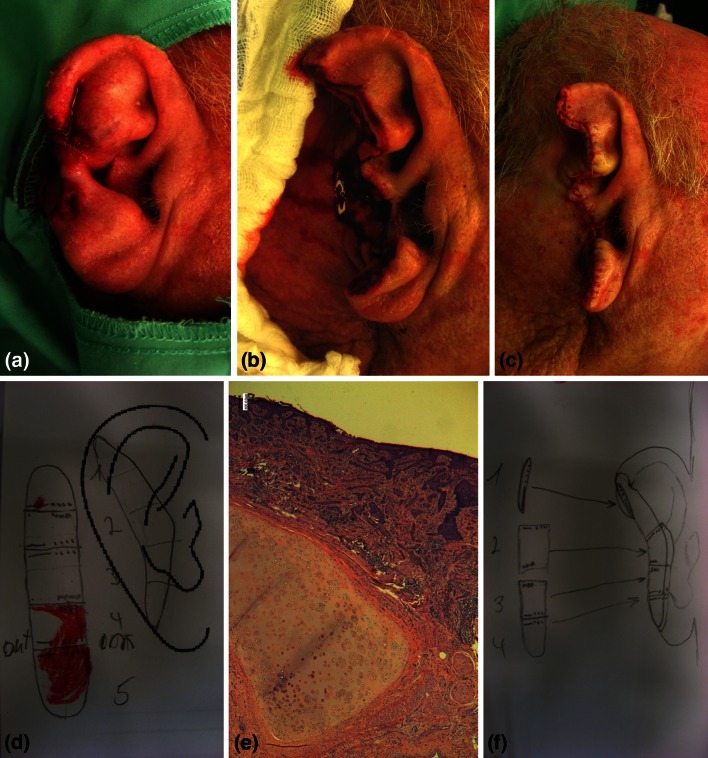
High-risk basal cell carcinoma on the ear. **a** Large sclerosing ulcerated plaque infiltrating and eroding the ear helix. A punch biopsy confirmed the presence of infiltrating basal cell carcinoma (BCC). **b** Final defect after 2 Mohs surgery stages and **c** side-to-side clousure. **d** Stage I Mohs map showing positive deep and lateral margins affected and **e** the ear cartilage (hematoxylin and eosin staining, original magnification ×40). **f** Stage II showed no residual BCC. Courtesy of Dr Zilinsky and Dr Bennassar

In contrast, relapsed aggressive forms of BCC benefit from a wide surgical management with the goal of achieving a complete resection, the best example of which is Mohs micrographic surgery (MMS) (Fig. 1). This complex surgical technique is focused on an extremely accurate assessment of margin status [18]. Achievement of margin-free of tumor invasion is crucial to avoid relapse either in high-risk or in locally advanced tumors. More specifically, MMS is a highly specialized microscopically controlled surgical technique aimed at removing complex or advanced skin tumors with poorly defined borders allowing histological examination of the entire surgical margin [19]. The Mohs surgeon should act as both a surgeon and a pathologist and should thus examine the microscopic margin status after removing the tumor. When used to treat patients with BCC, MMS is generally reserved for high-risk facial lesions (although not exclusively). As MMS is a very demanding technique, not all practicing dermatologists are well trained or have enough experience to conduct an MMS safely. However, the practice of MMS has clearly been rising in the last 15 years worldwide [20, 21]. Although strong evidence from randomized trials is lacking in the setting of BCC [22], one of the most important randomized trials performed to date in this setting addressed the use of MMS or surgical resection in facial BCC. Indeed, long-term results showed that MMS resulted in a lower rate of recurrences than surgical excision in the group of patients with relapsed BCC and differences in patients with primary BCC were non-significant [23]. Nevertheless, a consensus meeting sponsored by several academic institutions in the USA agreed on the appropriateness of the MMS approach for high-risk BCC located in different body areas [19]. In this consensus work, human body skin was split into three areas, H as high- (mask areas of face including central face, nose, eyelids, chin, ear, genitalia, hands, feet, nipples—areola, ankles), M as medium- (cheeks, forehead, scalp, neck, jawline, pretibial surface) and L as low-risk area (trunk and extremities, excluding H and M areas). In short, MMS was considered appropriate for almost every recurrent tumor and primary aggressive tumors. On the other hand, in patients with primary nodular tumors, performing MMS was considered appropriate for tumors sited at H and M areas and inappropriate (or of uncertain value) in most patients with tumors in the L area. Strategies including MMS aiming to manage special situations, such as tumors sited in previous irradiated skin, in traumatic scar, over an osteomyelitis, over an ulcer, over chronic inflammation and in patients with genetic syndromes, were all considered appropriate.

There are patients with high-risk tumors for whom management with local treatments such as surgical excision, MMS or radiotherapy may be very difficult and may lead to excessive morbidity and disfiguration. Moreover, achievement of tumor-free margins is difficult in these tumors. Therefore, these patients seem to be located on a blurry clinical boundary between high-risk and locally advanced BCC, which is also poorly defined from an academic viewpoint.

### Locally advanced BCC

Since BCC arises more frequently in the head and neck, the tumor may infiltrate the eye, nose, facial bones, skull or brain and result in significant symptoms and complications [24]. Thus, the tumor may invade the external auditory canal, auricular cartilage, temporal bone [25], base of the skull leading to cranial nerve palsies [26], brain causing central neurologic symptoms, face with cosmetic problems, calvaria [27] or eye leading to sight problems [28]. In such cases, treatment with surgery or radiotherapy can lead to the loss of sensory organs and their functions.

Although the best treatment for patients with locally advanced tumors is controversial, if the tumors are non-metastatic and local control may be achieved with surgery when technically feasible, this option should always be considered [29]. Thus, locally advanced tumors are managed, when feasible, with MMS aiming to achieve clear margins and ultimately the cure of the patient. However, this technique, used alone, may not be enough for treating these cases. Therefore, management of these patients may require very complex surgical procedures performed by multidisciplinary teams. These patients are challenging and thereby need an individualized and highly specialized approach. Table 2 summarizes treatment and outcome of selected cases of locally advanced BCC reported in the literature. Another option to consider in the management of these tumors is radiation therapy, alone or with salvage surgery if necessary [30, 31].

**Table 2:** Selected case reports of BCC locally advanced or metastatic with curative intent

Author, year (references)	Clinical summary	Treatment	Outcome/follow-up
Bozikov 2006 [46]	T in ear 3 cm; M1 cervical lymphadenopathy	Surgical resection with selective cervical lymph node dissection + RDT	No follow-up
Berlin 2002 [14]	T in back 3 cm; M1 axillary lymph nodes	MMS + axillary selective dissection	No recurrence after 18 months
Fantini 2008 [47]	T in axillary skin 1.5 cm, fixed	Wide resection + axillary lymphadenectomy	After 1 year local relapse; after 2 years bone and lung M1
Montgomery 2008 [48]	Multiple T in trunk	Preoperative radiotherapy→surgery	No follow-up
Majima 2012 [49]	T 3 cm in back	Surgical resection	After 4 years M1 supraclavicular and after lung M1
Wadhera A 2006 [10]	T 1.5 cm in ear	MMS	Local relapse after 5 years→MMS→ after 1 year parotid M1: resection and radiotherapy. No relapse after 2 additional years
Mencía 2005 [50]	80 years. T in lacrimal caruncle	Surgical resection	No recurrence after 7 years

*T* tumor, *RDT* radiotherapy, *M1* metastases and *MMS* Mohs micrographic surgery

The treatment options for those patients in which surgery is technically unfeasible were really limited until recently. Thus, a non-surgical approach to treat locally advanced BCC was sequential chemoradiation, which has been used as a treatment for giant locally advanced BCC. Cisplatin alone or in combination has been the most frequently used chemotherapy agent as part of these regimens [32]. However, chemoradiation treatment results in a transient control of tumor growth and ultimately the patient suffers tumor progression. Therefore, this strategy should be considered palliative.

### Metastatic BCC

The presence of multiple primary tumors in the region of the head and neck is one of the risk factors associated with the occurrence of metastasis [33]. Thus, between 85 and 90 % of metastatic BCC is due to head and neck primary tumors [10]. Metastatic BCC includes patients with distant metastases (bone, lung, liver) or lymph node involvement. The median overall survival for patients with metastatic disease is 8 months [9].

These patients require systemic therapy aimed at achieving an improvement in overall survival. Management of metastatic BCC with chemotherapy has not been widely used thus far. In spite of a very small number of reported cases experiencing a transient tumor response with platinum-based chemotherapy [34–36], there is general agreement in that the results of systemic chemotherapy are poor when treating these patients. Therefore, new treatment options are clearly needed, and to move forward enrollment of these patients in clinical trials should be encouraged.

### New therapeutic strategies

Locally advanced and metastatic BCC leads to severe complications secondary to important local tumor growth and can be life threatening. In addition, the QoL of these patients is severely impaired as a consequence of the morbidity, the local symptoms and the cosmetic impact of this disease. As we have outlined, results of the classic treatment are disappointing.

The periorbital area, as an example, is a special one because of both, the well-known predilection of BCC for this site and because cosmetic and functional concerns. Use of the MMS technique [37–39] or plastic surgery techniques with reconstruction [40] are options to treat these tumors with the goal of protecting the eyeball. As expected, incomplete resection is the main risk factor for recurrence of peri-orbital BCC [41]. Nevertheless, locally advanced BCC in this area may ultimately require orbital exenteration [42]. Therefore, the need of new therapeutic strategies and enrollment in clinical trials should be clearly underlined in the daily management of patients with locally advanced and metastatic BCC.

Recent advances in our understanding of the molecular pathways that are involved in the proliferation of BCC tumor cells have led to the development of new targeted therapies. The Hedgehog (Hh) pathway is abnormally activated in patients with both sporadic and inherited BBCs (Gorlin syndrome), and inhibition of this pathway appears to result in significant clinical responses. Mutations in PTCH1 (Patched 1) and SMO (smoothened) proteins of the Hh pathway appear to be the most common ones in BCC [43]. There are currently several novel Smo inhibitors (Table 3), one of which—vismodegib—has obtained FDA’s and EMA’s approval in 2012 and 2013, respectively.

**Table 3:** Hedgehog pathway inhibitors. Smoothened (Smo) receptor Inhibitors

Compound	Company
Vismodegib (GDC-0449)	Roche, Genentech
Sonidegib (LDE225)	Novartis
LY2940680	Eli Lilly
BMS-833923 (XLI139)	Bristol-Myers Squibb
LEQ-506	Novartis
TAK-441	Millenium Pharmaceuticals
Saridegib (IPI-926)	Infinity Pharmaceuticals
PF-04449913	Pfizer

To date, results from three clinical trials with vismodegib have been published [SHH3925g (phase I, *n* = 33), ERIVANCE BCC (phaseII, *n* = 104) and STEVIE (phase II, *n* = 1,229)] in metastatic BCC and locally advanced BCC [7, 8, 44]. Locally advanced BCC patients had cutaneous lesions that were larger than 1 cm and inappropriate for surgery (inoperable, multiply recurrent where curative resection deemed to be unlikely or for whom surgery would result in substantial deformity or morbidity) and for which radiotherapy was unsuccessful or contraindicated or inappropriate [8]. ERIVANCE trial showed overall response rates of 48.5 % in metastatic cohort and 60.3 % in locally advanced patients with a median duration of response of 14.8 months in metastatic BCC and 26.2 months in locally advanced BCCs [8, 45]. In the STEVIE clinical trial, an interim analysis in 500 patients with metastatic BCC or locally advanced BCC showed a response rate of 64.9 % [44].

## Conclusion

In summary, the standard of care for local treatment of high-risk BCC includes several options such as surgical excision, MMS or radiotherapy. When BCC progresses to an advanced state, in some cases lesions are no longer suitable for surgery, while in other cases surgery would result in substantial morbidity or deformity and systemic treatment is scarce. The inhibition of the Hedgehog pathway is a new strategy that challenges the actual and future options of treatment for metastatic BCC and locally advanced BCC patients.
